# Cytomegalovirus-Associated Inhibition of Hematopoiesis Is Preventable by Cytoimmunotherapy With Antiviral CD8 T Cells

**DOI:** 10.3389/fcimb.2020.00138

**Published:** 2020-04-21

**Authors:** Angelique Renzaho, Jürgen Podlech, Birgit Kühnapfel, Franziska Blaum, Matthias J. Reddehase, Niels A. W. Lemmermann

**Affiliations:** Institute for Virology and Research Center for Immunotherapy (FZI), University Medical Center of the Johannes Gutenberg-University of Mainz, Mainz, Germany

**Keywords:** bone marrow stroma, cytomegalovirus pathogenesis, graft failure, hematopoietic (stem) cell transplantation (HCT, HSCT), hematopoietic reconstitution, immunotherapy, murine cytomegalovirus, myelosuppression

## Abstract

Reactivation of latent cytomegalovirus (CMV) in recipients of hematopoietic cell transplantation (HCT) not only results in severe organ manifestations, but can also cause “graft failure” resulting in bone marrow (BM) aplasia. This inhibition of hematopoietic stem and progenitor cell engraftment is a manifestation of CMV infection that is long known in clinical hematology as “myelosuppression.” Previous studies in a murine model of sex-chromosome mismatched but otherwise syngeneic HCT and infection with murine CMV have shown that transplanted hematopoietic cells (HC) initially home to the BM stroma of recipients but then fail to further divide and differentiate. Data from this model were in line with the hypothesis that infection of stromal cells, which constitute “hematopoietic niches” where hematopoiesis takes place, causes a local deficiency in essential hematopoietins. Based on this understanding, one must postulate that preventing infection of stromal cells should restore the stroma's capacity to support hematopoiesis. Adoptively-transferred antiviral CD8^+^ T cells prevent lethal CMV disease by controlling viral spread and histopathology in vital organs, such as liver and lungs. It remained to be tested, however, if they can also prevent infection of the BM stroma and thus allow for successful HC engraftment. Here we demonstrate that antiviral CD8^+^ T cells control stromal infection. By tracking male donor-derived *sry*^+^ HC in the BM of infected female *sry*^−^ recipients, we show the CD8^+^ T cells allow for successful donor HC engraftment and thereby prevent CMV-associated BM aplasia. These data provide a further argument for cytoimmunotherapy of CMV infection after HCT.

## Introduction

The risk of cytomegalovirus (CMV) reactivation and consequent CMV disease remains a severe infectious complication in the treatment of hematopoietic malignancies by hematopoietic cell transplantation (HCT), which is the last therapeutic resort for aggressive leukemias that resist standard therapies. Accordingly, monitoring and management of CMV reactivation in latently infected HCT recipients is a medical task and challenge at transplantation centers worldwide (Seo and Boeckh, 2013; Ljungman et al., 2017, 2019; Stern et al., 2019). The aim of HCT is to repopulate the emptied bone marrow (BM) in HCT recipients who have undergone therapy-inherent hematoablative treatment for elimination of the malignant cells. BM repopulation requires homing of transplanted hematopoietic stem- and progenitor cells, here briefly referred to as hematopoietic cells (HC), to the stromal physical network of the BM. In so-called “hematopoietic niches,” the HC interact with stromal cells that provide essential growth and differentiation factors, the hematopoietins (Chabannon and Torok-Storb, 1992; Yu and Scadden, 2016; Lee et al., 2020). Successful engraftment reconstitutes cells of all hematopoietic differentiation lineages, including antiviral effector cells of the immune system.

Besides causing organ disease, such as interstitial pneumonia and hepatitis, CMV infection has been reported to directly interfere with the aim of HCT in that it inhibits myelopoiesis with the result of a graft failure (Simmons et al., 1990; Torok-Storb et al., 1997; Randolph-Habecker et al., 2002; Hancock et al., 2020). Notably, clinical isolates of human CMV differ in their tropism for HC and bone marrow stromal cells (Apperley et al., 1989; Simmons et al., 1990), so that the pathomechanism of impaired hematopoiesis likely differs between virus variants/strains. Cellular immunotherapy based on transfer of CMV-specific CD8^+^ T cells (Riddell et al., 1992; Walter et al., 1995; Moss and Rickinson, 2005; Feuchtinger et al., 2010; Neuenhahn et al., 2017) is an option to avoid myelosuppressive side effects as well as renal toxicity of antiviral drugs (Maffini et al., 2016) and to prevent CMV disease in those HCT recipients who are infected with drug-resistant CMV variants (Pei et al., 2017; Chemaly et al., 2019).

Recipients of solid organ transplantation (SOT) immunosuppressed for avoiding graft rejection by a host-vs.-graft (HvG) reaction are another group of patients at transplantation-associated risk of CMV reactivation and disease leading to graft loss by viral pathology. In both HCT and SOT, risk of CMV disease is rarely based on transmission of infectious virus from virus-shedding contact persons or with an acutely-infected transplant. Productive pathogenic infection rather results from the reactivation of latent virus under immunocompromising conditions, originating primarily from latently infected recipients' organs in the case of HCT and from latently infected donors' organs/transplants in the case of SOT (for a recent review linking latently infected cell types to reactivation risk in HCT compared to SOT, see Reddehase and Lemmermann, 2019). Notably, like in HCT, virus-specific T-cell therapy is a therapeutic option also in SOT, in particular to fight CMV variants that are resistant to antiviral drugs (reviewed by Roemhild and Reinke, 2016).

As clinical investigation into pathomechanisms is limited by exclusion of experimental approaches for ethical reasons, a mouse model of experimental HCT and infection with murine cytomegalovirus (mCMV) has been established to study the impact of a CMV infection on hematopoietic reconstitution (for a more recent review, see Reddehase, 2016) as well as to provide proof-of-concept for antiviral immunotherapy of lethal CMV organ disease by adoptive transfer of virus-specific CD8^+^ T cells in general (Reddehase et al., 1985, 1987a,b; for reviews see Holtappels et al., 2008; Reddehase and Lemmermann, 2018) as well as specifically in the HCT model (Steffens et al., 1998a).

Early work in a mouse model of incomplete hematoablation and mCMV infection has shown that the BM was replenished by day 14 in the absence of infection due to auto-reconstitution. In contrast, in the presence of infection, the initial endogenous hematopoiesis collapsed beyond day 6, resulting in BM aplasia by day 14 (Mutter et al., 1988). In accordance with cell culture data for the majority of tested human CMV clinical isolates (Apperley et al., 1989; Simmons et al., 1990), work in a cell culture model of murine myelopoiesis suggested that the cause of myelosuppression by mCMV is an infection of BM stromal cells rather than of HC (Busch et al., 1991). For testing this *in vivo* in a relevant clinical correlate, a sex-mismatched mouse model of HCT was established with HC from male donors, which carry the Y-chromosomal male-sex-determining gene *sry* (Gubbay et al., 1990; Koopman et al., 1990), and female *sry*^−^ recipients, or *vice versa*, so that either donor HC or recipient's BM stromal cells carry the *sry* gene as a reporter for donor or recipient origin (Mayer et al., 1997; Steffens et al., 1998b; Seckert et al., 2008, 2009). Collectively, the studies in the HCT model provided reasonable evidence to conclude that mCMV inhibits successful engraftment of donor HC in the stromal BM compartment of the recipients by downregulation of hematopoietin gene expression in stromal cells (Reddehase et al., 1992; Mayer et al., 1997; Steffens et al., 1998b).

Here, we have used this HCT model to address the obvious and medically relevant, but so far neglected question, if cytoimmunotherapy by adoptive transfer of virus-specific CD8^+^ T cells can prevent the infection of BM stromal cells of HCT recipients and thereby restore the stroma's capacity to support successful engraftment.

## Materials and Methods

### Experimental HCT and Infection

BALB/cJ (*H-*2^*d*^; MHC class-I molecules K^d^, D^d^, L^d^) mice were purchased from Harlan Laboratories and housed under specified-pathogen-free conditions at the Translational Animal Research Center of the University Medical Center, Mainz, Germany. Syngeneic HCT was performed in duplicate with 8–10 weeks-old male (*sry*^+^) HC donor mice and female (*sry*^−^) recipient mice (*n* = 5 per experimental group), essentially as described previously (Steffens et al., 1998b; Podlech et al., 2002). In brief, hematoablative conditioning of recipients was performed by total-body γ-irradiation with a single dose of 6.5 Gy from a [^137^Cs] source. At 6 h after irradiation, HCT was performed by intravenous infusion of 1 × 10^5^ HC isolated from femoral and tibial donor BM. Infection of the recipient mice with 1 × 10^5^ plaque-forming units (PFU) of purified mCMV, strain Smith ATCC VR-194, was performed subcutaneously at the left hind footpad at ca. 2 h after HCT (for a method book chapter, see Podlech et al., 2002).

### Adoptive Cell Transfer

A virus-specific cytolytic T lymphocyte (CTL) line specific for the D^d^-presented immunodominant viral epitope m164 (Holtappels et al., 2002; Fink et al., 2014) was generated by four rounds of restimulation of CD8^+^ memory T cells, which were derived from the spleen of latently mCMV-infected female (*sry*^−^) BALB/c mice, with the respective synthetic m164 peptide at a concentration of 10^−9^ M (Holtappels et al., 2002; Lemmermann et al., 2010). For cytoimmunotherapy of the infection, 1 × 10^6^ CTL were infused intravenously together with the HC.

### Quantification of Hematopoietic Reconstitution

Tibial and femoral BM cells were isolated from individual HCT recipients on day 14 after HCT. Total DNA was extracted with the DNeasy Blood & Tissue kit (Qiagen, Hilden, Germany). Quantification in absolute numbers of the *pthrp* gene (two copies in a diploid cell) and of the Y-chromosomal gene *sry* (one copy per cell) was performed by SYBR-Green qPCR (Lemmermann et al., 2010), normalized to a log_10_-titrated standard of linearized plasmid pDrive_gB_PTHrP_Tdy (Simon et al., 2005; Lemmermann et al., 2010). Note that testes-determining gene of Y (*tdy*) and sex-related gene of Y (*sry*), are synonyms (Gubbay et al., 1990; Koopman et al., 1990).

### Highly-Sensitive Quantification of Infectious Virus and Viral Genomes in Host Organs

The load of infectious virus in spleen, lungs, liver, and salivary glands of infected HCT recipients was assessed in the respective organ homogenates as PFU determined by virus plaque assay performed on mouse embryonal fibroblast (MEF) monolayers under conditions of centrifugal enhancement of infectivity (Kurz et al., 1997; Podlech et al., 2002; and references therein). The technique of centrifugal enhancement detects infectious virus with a ~20-fold higher sensitivity compared to a standard PFU assay with no centrifugation. Viral genomes were detected in organ homogenates as described previously (Lemmermann et al., 2010). Briefly, total DNA was purified with the DNeasy Blood & Tissue kit (Qiagen) and viral and cellular genomes were quantitated in absolute numbers by *M55* (*gB*)-specific and *pthrp*-specific quantitative PCR (qPCR), respectively, normalized to a log_10_-titration of standard plasmid pDrive_gB_PTHrP_Tdy.

### Quantification of Infected Tissue Cells and of Tissue-Infiltrating T Cells

The infection of the liver and liver tissue infiltration by adoptively-transferred antiviral T cells of the m164 epitope-specific CTL line (see above) was analyzed in the microanatomical context by 2-color immunohistochemistry (2C-IHC) specific for the intranuclear viral protein IE1 (red staining) and the membrane-localizing CD3ε component of the T-cell receptor complex (black staining), respectively. Infected cells in the BM stroma were detected after bone decalcification with trichloroacetic acid in thin-sections of sternum and vertebra by red staining of the IE1 protein (Dobonici et al., 1998). Sections were routinely counterstained with hematoxylin. Cell numbers are counted for representative areas of tissue sections (for detailed methods, see Podlech et al., 2002; Lemmermann et al., 2010).

### Statistical Analyses

To evaluate statistical significance of differences between two independent sets of data, the two-sided unpaired *t*-test with Welch's correction of unequal variances was used. In the cases of infectivity quantification and of cellular replenishment of BM, log-normally distributed data were log-transformed to enter the *t-*test. Differences were considered as statistically significant for *P*-values of < 0.05 (^*^), < 0.01 (^**^), and < 0.001 (^***^). Calculations were performed with Graph Pad Prism 6.04 (Graph Pad Software, San Diego, CA).

## Results and Discussion

### Transfer of Virus-Specific CD8^+^ T Cells Controls the Infection of Organs and Bone Marrow Stroma in HCT Recipients

The aim of HCT is to reconstitute cells of all hematopoietic lineages after hematoablative therapy. This takes some time, so that HCT recipients are transiently immunocompromised. Control of a CMV infection in HCT recipients depends on efficient and timely reconstitution of mature, antiviral CD8^+^ effector T cells. The mouse model of mCMV infection at the time of HCT was designed to mimic clinical cases of peri-transplantation reactivation of latent human CMV, with CMV disease/pneumonia becoming evident from 3 weeks post-HCT onward (reviewed by Seo and Boeckh, 2013). In our preclinical model, the peak of symptomatic lung infection after 3 weeks preceded the peak of lung infiltration by endogenously reconstituted virus-specific CD8^+^ T cells after 4 weeks, followed by resolution of productive infection (Holtappels et al., 1998). Depletion of CD8^+^ T cells during hematopoietic reconstitution after HCT leads to lethal multiple organ CMV disease (Podlech et al., 1998), including viral interstitial pneumonia (Podlech et al., 2000). Instead of waiting for endogenous reconstitution, transfer of mature antiviral CD8^+^ T cells can close the “window of risk” between HCT and completed reconstitution (for a review, see Reddehase, 2016).

**Figure 1:**
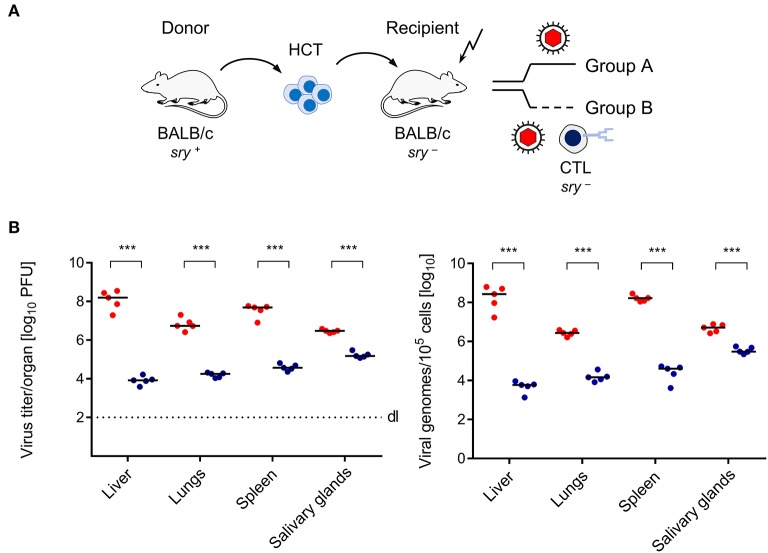
Control of CMV organ infection by immunotherapy with CD8^+^ T cells. **(A)** Sketch of the transplantation model defining group A (no therapy) and group B (therapy with virus-specific CTL). The flash symbol indicates hematoablative conditioning of the HCT recipients by total-body γ-irradiation. **(B)** Day-14 infection load in the organs of HCT recipients expressed as titers (plaque-forming units, PFU) of infectious virus (left panel) and as numbers of viral genomes (right panel). Symbols (red: group A; blue: group B) represent mice analyzed individually. Median values are marked. Differences between two experimental groups were determined by Student's *t*-test based on log-transformed, log-normally distributed data. Significance level: *P*-values of < 0.001 (***).

**Figure 2:**
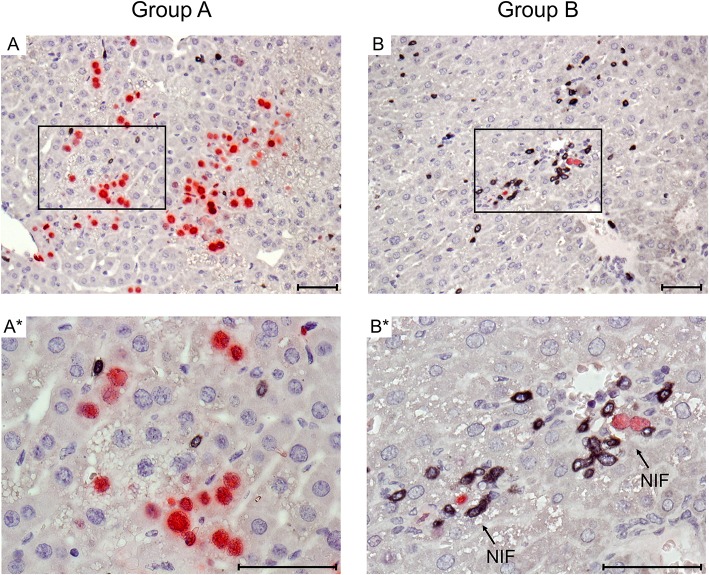
Immunohistological images of liver tissue infection and infiltration by T cells. Representative 2C-IHC images of liver tissue sections taken on day 14 after HCT and infection, corresponding to the infection load data shown in Figure 1B. (Red IHC staining) IE1 protein in nuclei of infected liver cells, which are predominantly hepatocytes. (Black IHC staining) CD3ε protein expressed by T cells. (Group A) No therapy. **(A)** Overview. (A*) Detail. (Group B) Therapy by transfer of CTL. **(B)** Overview. (B*) Detail. Frames in images **(A,B)** point to regions resolved to greater detail in images (A*,B*), respectively. Light counterstaining was done with hematoxylin. NIF (arrows in B*): nodular inflammatory foci that represent microanatomical structures where transferred CTL (black) congregate at infected liver cells (red) to prevent intra-tissue virus spread. Bar markers: 50 μm.

**Figure 3:**
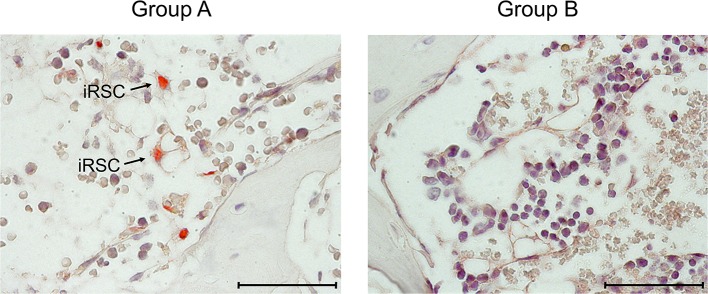
Immunohistological images of stromal infection in BM. Representative IHC images of vertebral BM sections taken on day 14 after HCT and infection. (Red staining) IE1 protein in nuclei of infected cells. Light counterstaining was done with hematoxylin. Arrows point to infected, very typically shaped “reticular stromal cells” (iRSC). (Group A) No therapy. (Group B) Therapy by transfer of CTL. Bar marker: 50 μm.

Here we have studied the mouse model of HCT and mCMV infection to test if transfer of virus-specific CD8^+^ T cells controls infection faster than endogenous reconstitution does, not only in the classical end-organs of CMV disease (Figures 1, 2), but also in BM stroma (Figure 3). HCT was performed with male (*sry*^+^) BALB/c mice as HC donors and female (*sry*^−^) BALB/c mice as recipients (Figure 1A). The recipients were conditioned by hematoablative treatment and infected with mCMV at the time of HCT. Group A recipients were left with no immunotherapy, whereas group B recipients received immunotherapy by transfer of *sry*^−^ antiviral CTL. Virus replication was quantitated on day 14, because group A recipients would die of multiple organ CMV disease and BM aplasia soon afterwards (Mutter et al., 1988). CTL specific for the m164 epitope of mCMV (Holtappels et al., 2002) were chosen because the epitope is very special in that it is encoded by transcripts of the viral Early (E) phase gene *ORFm164* as well as by a transcript from an upstream Immediate-Early (IE) phase gene (Fink et al., 2014; Renzaho et al., 2019). Accordingly, m164 epitope-specific CTL have the chance to recognize the corresponding D^d^-presented antigenic peptide on infected cells in the IE phase as well in the E phase of the viral replicative cycle, and this likely enhances the antiviral activity. In adoptive cell transfer models not involving HCT, protection against mCMV infection by m164 epitope-specific CTL has been shown repeatedly (Holtappels et al., 2002; Ebert et al., 2012; Nauerth et al., 2013). It was therefore not unexpected to find that the transferred cells reduced infectivity and viral genome load also in organs of the infected HCT recipients (Figure 1B).

Immunohistology was used to visualize protection against viral histopathology in the microanatomical context for the example of CMV hepatitis (Figure 2). mCMV infects hepatocytes and liver macrophages, also known as Kupffer cells, as well as liver sinusoidal endothelial cells (LSEC) (Sacher et al., 2008; Seckert et al., 2009; Lemmermann et al., 2015). On day 14 after HCT and infection, livers of group A recipients revealed a pronounced liver tissue infection and only a few randomly distributed, residual CD3ε^+^ cells. In contrast, in group B, transferred CTL were found to have congregated at very few remaining, infected liver cells to form “nodular inflammatory foci” (NIF). NIF are microanatomical compartments where CD8^+^ T cells recognize infected tissue cells and confine and eventually resolve the infection (Alterio de Goss et al., 1998; Holtappels et al., 1998; Podlech et al., 2000; Böhm et al., 2008). It is a frequently cited misconception that viral immune evasion proteins, which interfere with trafficking of peptide-loaded MHC class-I complexes to the cell surface (reviewed in Lemmermann et al., 2012), would completely prevent direct antigen presentation (Pinto et al., 2006). As we have shown previously for the example of the here chosen m164 epitope-specific CTL line, high functional avidity of T cell-target cell interaction is decisive for the recognition of infected cells and for *in vivo* antiviral protection in the presence of viral immune evasion proteins, whereas low avidity is only sufficient to recognize cells infected with a recombinant virus in which immune evasion genes are deleted (Ebert et al., 2012; Nauerth et al., 2013). All in all, the here transferred m164 epitope-specific high-avidity CTL clearly formed protective NIF and controlled the infection with wild-type mCMV, strain Smith, in organs of the group B recipients.

At this stage of investigation, it remained to be verified that the transferred cells also control the infection of BM stroma (Figure 3). On day 14 after after HCT and infection, which is a pre-final stage of lethal CMV disease in group A recipients, the stromal network was almost devoid of engrafted HC and their progeny, which defines BM aplasia. Notably, as indicated by intranuclear viral IE1 protein, infected reticular stromal cells (iRSC) could be detected. In contrast, iRSC were absent in the BM stroma of group B recipients after CTL transfer. Evidently, in this “therapy group,” the bone marrow was in a stage of ongoing repopulation.

### CMV-Associated Inhibition of Hematopoiesis Is Preventable by Transfer of Antiviral CD8^+^ T Cells

From the observed repopulation of the BM in the “therapy group” B, one may leap to the conclusion that the transplanted donor HC have successfully engrafted. However, previous work in a related model not involving HCT has shown that after incomplete hematoablation, auto-reconstitution takes place until day 6 in both uninfected and infected BALB/c mice before hematopoiesis breaks down in the infected group, while it proceeds in the uninfected group. Importantly, transfer of CD8^+^ T cells was found to rescue auto-reconstitution in the infected mice (Mutter et al., 1988; Dobonici et al., 1998). Hence, the possibility remained that prevention of stromal infection by the cell transfer rescued only hematopoiesis by residual stem cells, which have the advantage to have already occupied “hematopoietic niches” in the stroma, whereas transplanted HC may still fail to migrate to the BM and to compete for niches. This objection needed to be considered and addressed experimentally.

**Figure 4:**
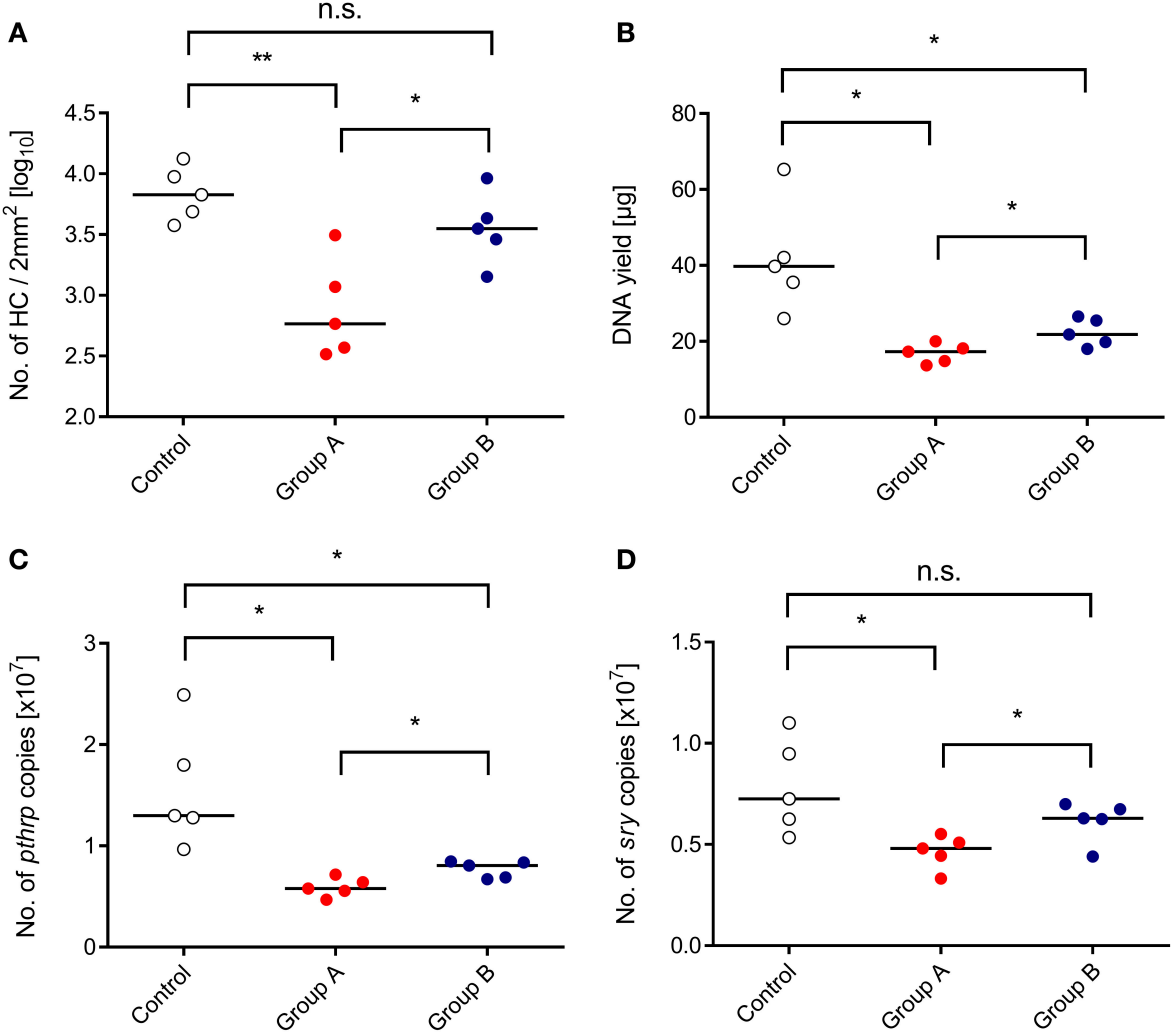
Inhibition of BM repopulation by CMV and its restoration by CTL transfer. (Control) HCT only. (Group A) HCT and infection. (Group B) HCT, infection, and immunotherapy with CTL. The indicated parameters of BM repopulation refer to day 14 throughout. **(A)** Hematopoietic cell (HC) counts refer to representative 2-mm^2^ section areas of sternal and vertebral BM. All other parameters **(B–D)** refer to BM cell yields pooled from both femora and both tibiae of individual mice. Symbols represent mice analyzed individually. Median values are marked. Differences between two experimental groups were determined by Student's *t*-test based on log-transformed data (upper left panel) or on non-transformed data (remaining panels). Significance levels: *P*-values of <0.05 (*), and <0.01 (**). n.s., not significant.

We approached this issue by transplanting HC from male (*sry*^+^) donor mice into female (sry^−^) recipients, and the CTL line was also derived from female mice (recall Figure 1A). As a consequence, quantification of the single-copy, autosomal gene *sry* by qPCR reflects the number of engrafted donor-derived *sry*^+^ HC and their progeny, whereas quantification of the single-copy, heterosomal gene *pthrp* by qPCR, divided by the factor of 2, reflects the number of all cells, which includes also *sry*^−^ stromal cells, transferred *sry*^−^ CTL, and *sry*^−^ hematopoietic lineage cells derived from a possible auto-reconstitution (Figure 4). All tested parameters, namely direct cell count in histological sections (Figure 4A), total DNA yield (Figure 4B), *pthrp* gene copy numbers (Figure 4C), and *sry* gene copy numbers (Figure 4D), were significantly lower in the infected HCT recipients compared to a control group consisting of uninfected HCT recipients. This finding reproduced the known inhibition of hematopoiesis by CMV. As already suggested by the BM repopulation visible in the histological images (Figure 3), all parameters were significantly higher in the “therapy group” B compared to group A. Importantly, quantification of the reporter gene *sry* proved that the rescue indeed applies not only to auto-reconstitution, but also to BM repopulation by the transplanted *sry*^+^ HC and their progeny.

In conclusion, adoptive immunotherapy of CMV infection in HCT recipients with virus-specific CD8^+^ T cells not only prevents CMV organ disease but also ensures successful engraftment of the hematopoietic transplant in the BM stroma of CMV-infected HCT recipients. As a research perspective, we propose that in the murine SOT model of kidney transplantation (Zhang et al., 2019), adoptive immunotherapy with antiviral CD8^+^ T cells will not prevent mCMV reactivation from a latently-infected kidney transplant but likely will prevent subsequent virus dissemination to recipient's organs, as already suggested by promising clinical data (Roemhild and Reinke, 2016).

## Data Availability Statement

The datasets generated for this study are available on request to the corresponding author.

## Ethics Statement

Animal experiments were approved by the ethics committee of the Landesuntersuchungsamt Rheinland-Pfalz according to German federal law §8 Abs. 1 TierSchG (animal protection law), permission number177-07/G09-1-004.

## Author Contributions

MR, JP, and NL designed the study. AR, JP, MR, and NL were responsible for the analysis and interpretation of the data. AR, JP, BK, and FB conducted the work and analyzed the data. MR wrote the manuscript. JP and NL were responsible for the data presentation. AR, JP, and NL contributed to the manuscript revision. All authors read and approved the submitted version.

## Conflict of Interest

The authors declare that the research was conducted in the absence of any commercial or financial relationships that could be construed as a potential conflict of interest.
